# Australians’ views on personal genomic testing: focus group findings from the Genioz study

**DOI:** 10.1038/s41431-018-0151-1

**Published:** 2018-04-30

**Authors:** Sylvia A Metcalfe, Chriselle Hickerton, Jacqueline Savard, Bronwyn Terrill, Erin Turbitt, Clara Gaff, Kathleen Gray, Anna Middleton, Brenda Wilson, Ainsley J Newson

**Affiliations:** 1 Department of Paediatrics, The University of Melbourne, Melbourne, Australia; 2 Genetics Education and Health Research, Murdoch Children’s Research Institute, Melbourne, Australia; 3 Sydney Health Ethics, Sydney School of Public Health, The University of Sydney, Sydney, Australia; 4 Garvan Institute of Medical Research, Sydney, Australia; 5 Genome.One, Sydney, Australia; 6 St Vincent’s Clinical School, University NSW, Sydney, Australia; 7 The Walter and Eliza Hall Institute of Medical Research, Melbourne, Australia; 8 Health and Biomedical Informatics Centre, The University of Melbourne, Melbourne, Australia; 9 Society and Ethics Research, Connecting Science, Wellcome Genome Campus, Cambridge, UK; 10 School of Epidemiology and Public Health, University of Ottawa, Ottawa, Canada; 11 Present Address: National Human Genome Research Institute, Bethesda, MD USA

## Abstract

Personal genomic testing provides healthy individuals with access to information about their genetic makeup for purposes including ancestry, paternity, sporting ability and health. Such tests are available commercially and globally, with accessibility expected to continue to grow, including in Australia; yet little is known of the views/expectations of Australians. Focus groups were conducted within a multi-stage, cross-disciplinary project (Genioz) to explore this. In mid-2015, 56 members of the public participated in seven focus groups, allocated into three age groups: 18–24, 25–49, and ≥50 years. Three researchers coded transcripts independently and generated themes. Awareness of personal genomic testing was low, but most could deduce what “personal genomics” might entail. Very few had heard of the term “direct-to-consumer” testing, which has implications for organisations developing information to support individuals in their decision-making. Participants’ understanding of genetics was varied and drawn from several sources. There were diverse perceptions of the relative influence of genetics and environment on health, mental health, behavior, talent, or personality. Views about having a personal genomic test were mixed, with greater interest in health-related tests if they believed there was a reason for doing so. However, many expressed scepticisms about the types of tests available, and how the information might be used; concerns were also raised about privacy and the potential for discrimination. These exploratory findings inform subsequent stages of the Genioz study, thereby contributing to strategies of supporting Australians to understand and make meaningful and well-considered decisions about the benefits, harms, and implications of personal genomic tests.

## Introduction

Since the mid-2000s, companies have been marketing various forms of personal genomic (DNA) testing to the public, typically online [1]. Termed by experts as direct-to-consumer genetic tests, they are marketed for a variety of purposes, offering a spectrum of information to consumers. This can include information about predisposition to conditions, carrier status, response to medications (pharmacogenomics), diet (nutrigenomics), fitness and sporting abilities, physical characteristics, ancestral connections and relationships, and for personality and behavior traits. Companies also market DNA testing for ageing, including skin ageing [2]; some even offer DNA dating tests, suggesting this could guide choice of partners [3]. Many ethical and social concerns have been raised and debated regarding personal genomic testing, primarily those tests available through the direct-to-consumer model [4]. The reliability and clinical validity of these tests vary considerably between companies, especially in terms of predicted risks for common complex diseases [5], while the scientific evidence for many of the more “recreational” tests, such as tests for sporting ability, are questionable [6]. Regulatory constraints in some jurisdictions have been imposed on the direct-to-consumer model, limiting some types of tests with health-associated results or requiring a health professional to request the online test on behalf of the consumer [7]. Current testing options are a combination of the health professional mediated and the direct-to-consumer model.

The healthcare system in Australia operates through a mix of publically funded (Federal and State) and private (user pays, but publicly subsidised) services. Traditionally, clinical genetics has been provided through publically funded services, while funding for clinical genetic testing has varied and, depending on the test and context, the patient may pay nothing or be required to partially or fully pay for the test themselves [8]. As healthcare costs rise and greater emphasis is placed on consumer choice and autonomy, genetic testing through the private sector is increasing,

Opportunities for Australian consumers to have direct access to personal genomic testing for health and non-health related purposes have been available, predominantly through online testing companies not located within Australia. The number and type of companies selling personal genomics tests (PGT) online (either directly or via a health professional) seem to fluctuate rapidly [1, 9, 10]. Direct-to-consumer genetic tests and in vitro diagnostic medical devices for health disorders were excluded from registration with the Australian Register of Therapeutic Goods in 2011 [11], thus apparently restricting access. This exclusion, however, does not extend to genetic tests for recreational purposes, such as ancestry, kinship, and parentage, and it is questionable whether this exclusion also applies to genetic tests around lifestyle and wellness, such as nutritional genomics.

As the field originated in the United States, most research examining experiences with personal genomics has been specific to that context. In Australia, there has been limited empirical research examining public [12–14] or health professional [15] views of, or experiences with, PGT. Findings from this earlier research reported minimal interest in pursuing such tests [13]. Within this increasing market, however, there has been little public engagement with Australians that details the expectations, benefits and drawbacks of undertaking these tests.

**Fig. 1:**
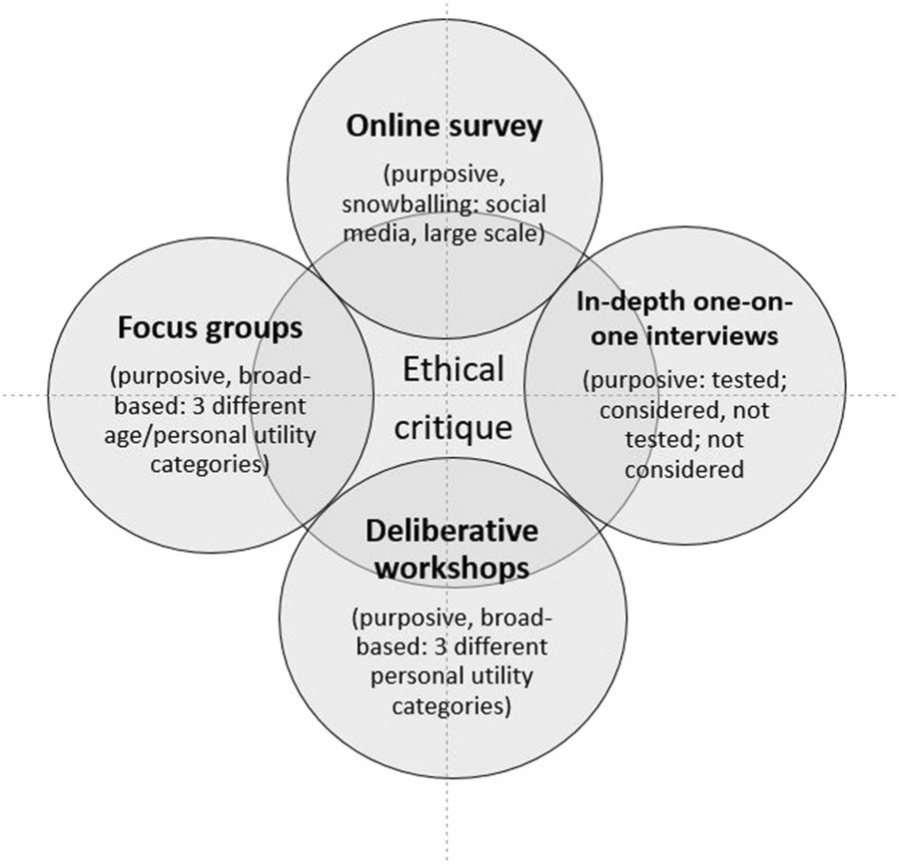
Overall design of Genioz study showing five research components. This paper presents findings from stage1, the focus groups

We are conducting a large multi-disciplinary and mixed methods study to understand the Australian public’s experiences and expectations of personal genomics. Genioz (Genomics: National Insights of Australians) commenced in 2015 and comprises five research components (Fig. 1). We are identifying important gaps in critical thinking that may undermine informed uses of personal genomics in Australia, to generate recommendations about educational and public engagement strategies to both support the public to make well-considered and meaningful decisions, and to advise policy makers regarding how the public might be supported accordingly. The first exploratory stage of the study is reported here. It used qualitative focus group methodology to collect baseline data around Australians’ awareness, knowledge, attitudes, and views on PGT.

## Participants and methods

### Ethics approval

Approved by The University of Melbourne Human Research Ethics Committee (ID 1543685).

### Recruitment

Recruitment was designed to target non-expert members of the public, using a variety of approaches to notify people about the study (Supplementary material Table S1). Potential participants contacted the researchers via email or telephone to register their interest. Researchers followed up with potential participants who were invited to take part in a focus group at a time and day that suited the majority. Focus groups were held in a variety of community-based locations in Melbourne (Victoria) and Sydney (New South Wales) that were accessible via public transport or car. At completion of the focus group sessions participants received an AUD$25 gift voucher and those who had travelled >50 km were compensated for their travel costs.

### Focus group data collection

Focus groups provide a useful approach for gathering qualitative information about collective views and meanings underlying those views, permitting interactions between participants, and for exploring a topic to be used in later stages of a study [16, 17], which is the case in the Genioz study. The recommended size of focus groups varies, but usually comprise between five to eight participants [16, 17]. During 2015, focus groups were conducted with participants grouped by age (18–24 years, 25–49 years, and ≥50 years). These age groupings were sampled purposively to anticipate likely differences in life circumstances, and therefore potential interest in different purposes of testing, such as fitness, planning to have children, ancestry, with two focus groups per age category planned. Participants completed the consent form and socio-demographic information: age, gender, relationship status, education levels (in general and with regard to genetics), and their experience with genetic conditions personally or through family/friends.

All focus groups were facilitated by SM, assisted by two note takers, and were audio-recorded. The focus group schedule followed Krueger’s format: starting with a “round-robin” open question to engage all participants, then with transition questions, ending with key focused questions [17]. Following the round-robin, the facilitator gave a very brief presentation introducing the concept of personal genomics and direct-to-consumer testing, with examples showing the home pages of websites for a few types of testing (ancestry, fitness, health, and lifestyle), but there was no other information presented such as pros and cons. General topics discussed in the focus groups included: perceptions of genetic concepts and how these arose; situations in which PGT are used; discussions around the benefits and harms of PGT; and participants’ interest in PGT (see Supplementary material Table S2 for schedule of questions).

### Data analysis

Focus groups were transcribed verbatim, checked for accuracy of transcription, and de-identified. Transcripts were imported into NVivo 10 (QSR International Pty Ltd, Melbourne, Australia) for data management. The analysis undertaken was qualitative description, however, since the round robin questions were directed and answered by everyone it is possible to make some semi-quantitative assessment of participants’ responses. This was done using manifest content analysis [18]. Thematic analysis was performed on discussion of the open-ended questions to develop codes using the approach described by Braun and Clarke [19]. Analysis was conducted independently by researchers CH, JS, and SM, and was concurrent with data collection that allowed for further question prompts as concepts emerged. Themes were derived inductively and compared iteratively within and between focus groups using the constant comparative approach to generate descriptive codes [19]. Quotes are presented either in text or in tables to illustrate themes.

## Results

### Participants

Altogether, a total of seven focus groups with 56 participants were held in Australia between July and September 2015: three in Sydney and four in Melbourne. An extra focus group was held in Melbourne in response to the high demand for participation in the oldest age group. Since the intention of the focus groups was to gather exploratory information and provide insights to inform the next stage (the survey), recruitment until saturation of themes occurred was not the purpose, although in fact no new concepts were emerging by the end of data collection. A summary of the participant sociodemographic data can be found in Table 1. About three-quarters had completed or were currently attending university and just under half had studied genetics at high school or at a higher level, with this being highest in the youngest age groups. University students accounted for about a third of the sample, a third were retired, and the remaining third were working in a variety of jobs.

**Table 1:** Socio-demographics of participants in focus groups

Ages of participants (years)	Focus group	Total *n* (%)	Males *n* (%)	Females *n* (%)	University education (current/completed) *n* (%)	Studied genetics formally (at high school or higher) *n* (%)	Children *n* (%)	Genetic condition (person/family) *n* (%)	Had genetic test (person/family) *n* (%)
18–24	FG1 M^a^	10	5	5					
	FG7S^a^	6	2	4					
	Total	16 (28.6)	7 (43.8)	9 (56.2)	16 (100.0)	12 (75.0)	0 (0.0)	0 (0.0) 3 unsure	0 (0.0) 3 unsure
25–49	FG3M	9	5	4					
	FG5S	5	2	3					
	Total	14 (25.0)	7 (50.5)	7 (50.0)	9 (64.3)	5 (35.7)	4 (28.6)	4 (28.6) 4 unsure	2 (14.3) 4 unsure
54–80	FG2M	9	2	7					
	FG4M	11	4	7					
	FG6S	6	2	4					
	Total	26 (46.4)	8 (30.8)	18 (69.2)	17 (65.4)	8 (30.8)	22 (84.6)	5 (19.2) 6 unsure	5 (19.2) 3 unsure
Total	7	56 (100.0)	22 (39.3)	34 (60.7)	42 (75.0)	25 (44.6)	26 (46.4)	9 (16.1%) 13 unsure	7 (12.5%) 10 unsure

^a^FG is the abbreviation used for focus group; the number represents the order in which the focus groups were conducted; M represents Melbourne and S represents Sydney, the two cities in which the focus groups were held

### Themes

#### Articulating genetics: explaining concepts, with minimal terminology

Participants’ levels of awareness and understanding of genetic concepts varied greatly across the focus groups. When asked what immediately sprung to mind when the words “DNA” and “genes” were mentioned, there was a wide range of responses and representations (Supplementary material Figure S1), but mostly participants spoke about: “genetic makeup” or “blueprint” that makes them “unique” and what makes a “human” who they are; “heredity”, traits that are “inherited” from parents and grandparents, or are “passed on” to children; and they often mentioned “genetic testing”, for a variety of purposes but predominantly health. A few participants across the range of focus groups referenced “Watson and Crick”, and used specific terms such as “double helix” and “chromosomes”. Apart from those individuals studying biological sciences at university, younger age groups did not generally demonstrate a greater knowledge of genetic concepts, even though they would have had greater and more recent exposure to genetics teaching at school.

Apart from those who had recently studied genetics at university, many participants had not previously heard of the term “personal genomics” but were easily able to infer its meaning:

“…it’s not a term I’ve heard before but it sounds like, when you put the word ‘personal’ in front of it, it makes me think oh, this is something directly related to myself and my own genetic code or my own genetic makeup” (FG3M, 21).

When also asked who was familiar with the term “direct-to-consumer” genetic testing, very few people had heard of this, although in one focus group there was discussion about what it meant:

FG5S-42: “Because you can buy it straight from the manufacturer? Rather than going through…”

FGS5-44: “That was my understanding.”

FG5S-42: “Like having to go to a doctor or something like that and then they give you the test.”

In the discussion that followed the brief explanation of personal genomics and examples of testing websites, many participants were able to articulate concepts around the contribution of genetics to physical characteristics, health, behaviors and disease, and the extent to which they perceived genes have a role. Physical characteristics were often considered to be inherited, with genes playing an important part, as they did with sporting abilities and some other talents, although some were sceptical that genetic testing could predict a person’s range of abilities (Table 2: Quotes 1–4). While participants discussed to what extent they believed genes contribute to health and disease, this was tempered by consideration of the impact of lifestyle and environment. Participants typically raised the “nature vs. nurture” concept, with some talking about genes predisposing to aspects of health and disease (Table 2: Quotes 5–8); however, there was no clear consensus around the relative influence of genetics on talent, personality, behavior, health, or mental health (Table 2: Quotes 8 and 9).

**Table 2:** Quotes illustrating theme 1: Articulating genetics: explaining concepts, with minimal terminology

Quote number	Participant ID	Quote
1	FG5S-44	“In terms of physical appearance, I think that’s determined by genes.”
2	SM FG6S-44 FG6S-42	“What do people think about [role of genes in] sporting ability?” “Rubbish.” “Sporting, well I think that runs in families, you know. I didn’t get the gene, but [laughter]. My grandfather was a sportsman, and his children were sporty. It runs in the family and one of my, one of the branches, they had a lot of success in sport, so I can see it passed on.”
	FG6S-43	“There wouldn’t just be one though, there would be like, thousands. And I assume they interact with each other, so, I think you’d be, you know, really shooting in the dark.”
	FG6S-40	“To claim that you could read it and predict it.”
	FG6S-43	“Yeah, it would be a bit absurd, I think.”
3	FG4M-35 SM FG4M-35 FG4M-31	“And I also think there are other abilities that get passed down through…” “What sort of abilities?” “Um, writing, those literary sort of things.” “Musical abilities.”
4	FG6S-46	“They [genes] predetermine, like appearance, but, how can you look at a gene and say yes, he can play the violin or he can play the piano. How can that be?”
5	FG2M-11	“Genetics are only part of the story, you know its nature/nurture, like we can be given a set of genes but that’s not the end of the story, it’s how we use our bodies and how we develop our bodies so it’s not all dependent on genes but having said that if you were to have the *BRCA* gene for breast cancer or the diabetes gene…”
6	FG3M-27	There’s a nature vs nurture debate so you have your DNA and that gives you propensities to have certain things, but how the environment affects your DNA, you can’t really tell, so say you have heart disease in your family, you may not actually get it even though you have mutations that make you more likely to get it. So, and there are so many genes that are controlled by environmental factors so, if you have the right environment, they get turned on or turned off. So, it’s not just DNA.
7	FG7S-52	“Coming from a non-science background before hearing what you were saying, um, I would have said genes definitely contribute somewhat, especially to inheritable diseases and just general health and well-being I think. But, it’s also a lifestyle thing, um, again, education is really, really important, like access to healthy food, like access to education on the importance of movement and exercise, I think that makes a huge contribution to your lifestyle and health.”
8	FG1M-9	“…breast cancer that runs through families and the genetic tied diseases umm, but other things I think, you know, the idea that your mum or dad had depression so you’ll probably have it too because it’s genetically tied, I think that’s a bit more environmental than, necessarily like, 100% oh, your mum was depressed so you’re going to be depressed as well.”
9	FG3M-20 FG3M-25	“I think like personality, or peoples’ mood, emotional response might have something to do with DNA or genes, like if the parents are easily get depressed or they’re happy, always very happy, the children might be similar.” “I disagree, because the depression situation also depends on the environment and the lifestyle factors, food plays a very major role in depression and also the situation, family situation.”
10	FG1M-6	[Discussing genetic testing] “Looking at epigenetic modifications as well, like…methylation”
11	FG2M-11 FG2M-13 FG2M-15 FG2M-11	“I have only…surface information but one of the studies that was done was on identical twins that had 98% the same gene makeup when they were babies, I think up until 3 years old, but when they turned 50 they had less than 3% because even though they were identical they were brought up the same, different things that had happened you know.” “Yes the effects.” “Influences.” “Different features came on and off, one had got very badly freckled and the other didn’t.”
12	FG3M-27 SM FG3M-27 SM FG3M-25 FG3M-27 SM FG3M-27	“Well, they’re basically genes that are affected by your environment, so the environment does cause changes in the genetic code which does… “So I’ll clarify that, it doesn’t actually change the genetic code.” “Well not the genetic code, but it does change how protein production happens.” “Yes.” “It mutates.” “Not mutates.” “No.” “It turns it on or off, switches.”
13	FG3M-28 FG3M-21 SM FG3M-21	“There was a headline in the [newspaper] I think about holocaust survivors passing on the trauma that they’ve been through when their, their children’s DNA is actually influenced by, I’m assuming that’s epigenetics.” [further discussion] “But definitely like there was a [newspaper] article recently that was on a study that was done over you know, yearly gaps with twins, and I’m a twin so, it just naturally piqued my interest. So I went to read this study and it was all about nature versus nurture with these twins…. it was just a very basic study, and it was really interesting, and of course the twins did both genetically change or alter the depending on what had happened. “ “So that’s epigenetics.” “Yeah, there you go, so I know about it, I just didn’t know.”
14	FG4M-32	[Discussing genetic screening in newborns] “But in any case then the role of epigenetics, is taken as being irrelevant. If you are reading those, reading that DNA information when the child is just a few days old. You’re saying that’s casting a die that epigenetics has no role in.”

In discussing the effects of environment, many alluded to how it might impact upon a person’s genetic makeup. A few discussed that environment might change genetic sequences or structure, while others talked about “switching genes off and on” (Table2: Quote 6). In four focus groups, at least one participant in each mentioned the term “epigenetics” without prompting, although most had never heard of this specific term. Some correctly described its effects without knowing the term itself, however there was some confusion in the discussion reflecting limited understanding of how epigenetic changes actually affect gene expression (Table 2: Quotes 10–14).

#### Building a framework of understanding of genetics and assessing legitimacy of information

Participants’ understanding of genetics appeared to come from varied and multiple sources, and evolved over time. The four most commonly mentioned categories of sources of information were: news media; searching the internet for information; from their formal education; popular science sources (Supplementary material, Figure S2). Other stated sources included: discussions with family, friends, through social media, or with a health practitioner, perhaps via a discussion of family history and traits within families; original research articles in journals; science fiction movies. Participants also discussed their perceptions of the legitimacy of these sources and considered some more reputable than others. There was often scepticism about science news that was portrayed in a sensational way, through various forms of media including online (Table 3: Quotes 1 and 2). Every focus group discussed the importance of authoritative sources of information, some even mentioning checking back to the original “evidence” and “peer-reviewed” research (Table 3: Quotes 3 and 4). There was a range of opinions around what constitutes a reputable and authoritative source of information. Some thought that it was important to understand the motivations of those providing information, expressing cynicism regarding commercial motivations (Table 3: Quote 4). In one focus group of younger people, there was some discussion about how information could be considered more credible when mentioned by more than one source or person, even if those sources may not always be regarded as expert (Table 3: Quote 5). Nevertheless, many felt that information provided by independent academic, medical and government funded health agencies would have more legitimacy (Table 3: Quote 6), although pronouncements from government via politicians *per se* might not carry the same weight (Table 3: Quote 7).

**Table 3:** Quotes illustrating theme 2: Building a framework of understanding of genetics and assessing legitimacy of information

Quote number	Participant ID	Quote
1	FG3M-21	“When it comes down to genetics and DNA…I feel very sceptical whenever I approach something in the mass media way because the general gist of what they’re trying to do is be sensational about it. So that will be like, ‘they’re going to change your genetics and we’re going to have four heads’ and so then everyone like snaps it up and reads it, so there is a scepticism about that, but I like getting different points of view from lots of different places so yes, we are exposing ourselves to it on social media all the time so there are different articles and you can go and look at different viewpoints…”
2	FG5S-43	“…and the other thing is, I kind of have two baskets, a crap basket or a fact basket. And most of the stuff in the media goes into the crap basket.”
3	FG4M-29 FG4M-36	“I think an authoritatively written piece in a reputable source with preferably some reference to peer-reviewed research you’d have reasonable confidence in…” “You’ve got to be sceptical at times by what you read in newspapers because sometimes it just goes against what you already know, you know yes there could be a paradigm shift and they’ve moved somewhere else but I think you’ve got to get back to the basic research to find out what really is the case.”
4	FG5S-43	“The main thing that I look for… in looking for evidence is, particularly, if you’re talking about genetics, is, what’s the motivations of the people with actually putting information out there? What’s their, what’s their goal? Where do they get the funding from? Are they trying to sell something? So, it goes through those type of filters first, that, that level of cynicism, because not everything is altruistic. In fact, quite a lot isn’t…”
5	FG1M-5 SM FG1M-5 FG1M-4	“I don’t really read the newspaper, I’m more on the internet and stuff… I read it, but I don’t…believe, and if… it really interests me then I’ll put further effort into…looking it up and stuff, so if I…think it has value then I’ll search it into more detail [online]”…”I’ll check other sources, so I check… if it’s just one source then I’m like ooh, and then if there’s a couple of people saying it then I feel like it has some kind of value to it.” “So if more people say it, you’re more likely to believe it?” “Yeah…” “…yeah, I feel like if multiple people are sort of saying the same thing you have to sort of take it for what it is in a sense and if you’re really that concerned about it then go and talk to someone [expert]…”
6	FG3M-28	“I might go and get more detail from another place, find something with a dot ‘edu’ in it.”
7	FG6S-46	“Reputable…organisations, like via the Internet…. A government agency, yes, but, not, not government.”

#### Forming perceptions of the role of personal genomic testing

There were diverse views about the value of undergoing personal genomic testing and the reasons that might motivate people in general. Many could see the usefulness of testing that is health-related, particularly for conditions where treatment or prevention can be used (Table 4: Quote 1), and having information from testing to allow them to prevent or avoid passing on genetic conditions to their children (Table 4: Quote 2). Some were also concerned with the psychological impact of knowing this information (Table 4: Quote 3). For some, this perception of utility extended to considering benefits around testing and nutrition (Table 4: Quote 4), as well as response to medications (Table 4: Quote 5), but more rarely around testing for fitness, which was mentioned by just a few participants in the two youngest age groups (Table 4: Quote 6). Others thought that testing out of curiosity alone had its merits (Table 4: Quote 7), and a few older participants were interested in DNA testing around ancestry (Table 4: Quote 8). However, even when discussing health-related testing, people expressed reservations about how advances in technology are driving the direction of where testing is heading (Table 4: Quote 9). Participants expressed even greater apprehensions and reticence when considering testing and possibly selecting for characteristics that are less health-related (Table 4: Quotes 10 and 11 dialog).

**Table 4:** Quotes illustrating theme 3: Forming perceptions of the role of personal genomic testing

Quote number	Participant ID	Quote
1	FG5S-44	“…if information can come out of testing that you’re… predisposed for having cancer or a particular illness, then I would imagine … that’s good information to then … go back and take preventive measures or be kind of conscious of what can happen, in your health down the track.”
2	FG3M-27	“If you knew there was something quite serious that runs through your family, the probability of passing it on, if there’s a genetic testing … genetic counseling kind of stuff, I’d possibly be interested but I mean, I suppose if it’s really debilitating now.”
3	FG1M-4	“…the testing [a positive thing], if you were prone to have breast cancer … it’s good to go for check-ups and … if you wouldn’t know, you would go for check-ups and things like that, but then there’s bad things, like I see the anxiety that would follow if you were told, maybe it would always be in your mind.”
4	FG5S-43	“I’d be interested in knowing about the nutritional things. Because I have always had an issue with my weight …but, if I knew, at a young age, that I had a disposition to, uh, be overweight, or, I had an increased risk of heart attack, or, diabetes, or, any of those serious health issues, um, maybe my parents would have brought me up differently, or maybe my diet would have been different, maybe I wouldn’t be overweight right now.”
5	FG6S-48	“If I was diagnosed with cancer and the oncologist said, I want to prescribe you this [drug] but I’m not sure, I’d say yeah, go ahead and test it.”
6	FG7S-52	“…health wise, the only other thing I would be interested in would be fitness and nutrition, cause that is something I’m personally interested in. But what I’m doing right now works for me.”
7	FG4M-38	“Just knowledge, to contribute to our understanding of what the universe is about, … and what our life has come from, don’t you find that as curiosity…”
8	FG6S-43	“…ancestry, curiosity, would, would be, you know, you might have been a Viking, you never know…”
9	FG6S-49	“I don’t know, I just feel very uncomfortable about it because, I mean, with some of those conditions, yes, I don’t have a problem about it, but just because the technology is there, doesn’t mean to say we should be using it. Because it means that technology is driving our values in a way, and, I’m not quite sure that I particularly like that, although, got to say that technology rules our lives anyway, with television and cars and trucks and whatever else, so… “
10	FG1M-6	“I just don’t like testing for all these other things that are coming, like eye color and hair, I just don’t want to go there.”
11	FG3M-28 FG3M-23	“I wasn’t aware that science was getting so advanced now that we can unlock DNA and see what everything is and tell what a person’s life is going to be by looking at the genetic code, you know, I’m sort of hoping we’re not getting to that degree, but also I’m nervous that we’re going to start tampering with genetics and I’m nervous that there’s going to be designer babies, I’m nervous that there’s going to be sexuality decisions made by parents, I’m nervous that there’s going to be parents who want the perfect children.” “I’ve heard of the designer baby thing already where they … a couple can’t conceive so they do the artificial insemination, and they create four embryos and they just pick the best ones, so we’ve already gotten to the point where they can just…”

#### “If the need arose”: Considering personal genomic testing for oneself

Although a number of participants reported having had a genetic test themselves or within their family, these were predominantly in the context of clinical genetic tests ordered by a medical professional. Only one person reported having had a personal genomic test accessed directly online.

When participants were asked to consider whether they would undertake personal genomic testing themselves, views were varied. More than half of participants said they would *consider* having testing, although very few participants reported they would be keen to have PGT. Those who were interested appeared motivated by curiosity and a desire to contribute to science. However, this interest was qualified and focussed on health information that was actionable and especially if relevant to current or future children and other family members:

“…health things, but only if there was … cures for, you know, it was positive. There’s no point in finding out you’ve got something that can’t be fixed.” (FG6S-46)

Many felt testing should be prompted by a reason, for example, impending ill health, or if recommended by a health professional or a health campaign from non-commercial sources.

“I think if the need arose or the reason arose I’d be quite happy to have it. Unless the need arose, I don’t think I’d do it for any other reason, and certainly [would] for my family.” (FG2M-18)

For those less enthusiastic about purchasing a test for themselves, the main concerns raised were cost, perceived lack of validity and/or utility of the results, and worries about potential impact on privacy, insurance and discrimination.

“I’d definitely make sure I got it done from a reputable person, it, yeah, cost would be a major factor though, obviously. If it was within like a significant amount of money, I’d definitely think quite hard about it.” (FGS7-51)

FG4M-30: “But I see in the future that life insurance companies will be probably demanding this.”

FG4M-32: “That’s a problem.”

FG4M-38: “I think they do in America already.”

FG4M-30: “Oh they do it already do they?”

FG4M-29: “One of the things I find most worrying about it.”

SM: “So are you all a bit worried about insurance?”

FG4M-32: “Yeah, absolutely, misuse.”

FG4M-30: “Well for all this it’s privacy isn’t it? Who owns this information?”

In general, participants expressed a general sense of distrust and scepticism about the marketing rhetoric used in the websites and the commercial interests of the companies offering testing.

“Yeah those websites, oh this is about making money, I would be dubious and certainly from my experience on the Internet you get a bit of a sniff detector, you can pick up when something feels a bit bogus, fairly quickly.” (FG2M-13)

## Discussion

These qualitative focus group findings are the first to be reported from a larger mixed-methods, multi-stage study on Australians’ perceptions and expectations of personal genomic testing—the Genioz study. Awareness of personal genomics was fairly limited across the focus group participants. Their level of knowledge and understanding of genetics and genomics appeared to be drawn from several sources; these included news and popular science media, formal education, experience with genetic testing and/or genetic conditions or personal interest. Overall, participants did not think in deterministic terms, acknowledging that the environment played a role in a person’s characteristics including health. While there was interest in testing for health and ancestry testing, many participants expressed scepticism about the types of PGT available and what might be done with that information.

Regardless of the earlier research in Australia, the general lack of awareness of the availability of genetic tests via the internet by our focus group participants, apart from those who had studied this topic at university, does not reflect the increasing level of media attention to the rapid pace at which genetic technologies are being introduced and their potential applications [20]. In the advertising material used to recruit for the focus groups, we elected simply to refer to genetic testing for the variety of purposes (e.g., ancestry, fitness, health and risk of disease) rather than how the testing is accessed. We then introduced the latter in the brief information we presented to the groups, by showing several websites of testing companies, and saying that these types of tests are available on the internet (with or without the involvement of a health professional). In Australia there were over 12.8 million internet subscribers in 2015, with 86% of Australian households having internet access at home [21], and 61% using the internet to obtain goods and services [22], suggesting that Australians are clearly potential consumers in this space.

Terms from the academic literature—“personal genomics” and “direct-to-consumer” genetic testing—were not known to the majority of the participants. Even before the discussion about personal genomics, however, participants also spoke about DNA and genes as being “unique” to an individual, underlying the link participants were able to make with “personal genomics”. A number of participants referred to TV programs around DNA forensics in crime scene investigations, which centers on the uniqueness of a person’s DNA sequence and may have contributed to their beliefs. Testing companies often refer to “DNA testing”, “genetic testing”, and more recently “personal health genomics” and “personal genome service” on their websites; the term “direct-to-consumer genetic testing” or DTC-GT is not used by the companies themselves, yet has been ascribed almost universally to this type of testing in the academic literature [5, 23, 24]. Indeed, guidance about DTC-GT aimed at the public, researchers and health professionals, in the form of policies, position statements and recommendations by professional bodies [25], including Australian [26–29], almost invariably include “direct-to-consumer” or “marketed directly to consumers” in their titles. Furthermore, the term itself can be considered misleading as it now encompasses tests that are marketed directly to the consumer but not necessarily ordered by the consumer *per se* if a health professional is required to order on their behalf. Thus, there seems to be a disconnect between the language used in scholarly publications leading to policy recommendations, and that used by the testing companies and the public. This raises the question of whether the Australian public would be able to search for and find these guidance documents if this term is not part of their lexicon. More recent USA literature refers to “personal genomics” [24] and “consumer genomics” [30]. Based on our findings, recommendations and educational support should use language more accessible to the public who might be searching for such information; indeed, we would suggest that future guidelines avoid the use of the term “direct-to-consumer” completely in their titles.

Similar to other studies [31], participants in the focus groups generally articulated concepts of genetics through the lens of heredity/inheritance rather than in molecular terms or mechanistically (Supplementary Figure S1). Participants spoke about the contribution of genetics to characteristics and disease, and appreciated the role of genes without subscribing to genetic determinism. As discussed by Condit [31], there was the tendency to lend more weight to genetic contribution to physical characteristics than to, for example, mental health conditions, although this was a matter of debate among the focus groups. Despite their general lack of familiarity and use of specific terms, participants’ conceptual understanding of the interplay of genes and environment was reasonably sophisticated. Certainly the role of the environment acting through epigenetics was a recurring topic. But there was clearly some confusion about underlying mechanisms of environmental effects; that is, whether the effect is due to the environmental agent causing mutations in the DNA sequence, or whether the effect is through epigenetic “gene silencing” mechanisms.

Focus group participants were relatively well-educated participants who built their understanding of genetics and genomics from a range of sources. There was discussion about the legitimacy of information sources, with some examples they construed to be authoritative. For some it was important to validate information they came across in the news media by searching original research articles, while others were more accepting of information at face value. However, one aspect of the discussion that emerged from a younger group was their perception that information they encountered multiple times seemed more credible, irrespective of its source. The tendency to follow others’ opinions has increased among Internet users, and this “bandwagon” effect has been noted amongst Internet users who are not motivated enough by a particular topic to systematically seek information, but instead peripherally judge or process the abundance of data available to them through social media with minimal effort, using a range of cues [32]. In terms of wanting information about PGT, participants preferred that this should be provided by independent academic and health agencies and were sceptical of receiving this from politicians or from companies themselves, which they felt were driven by commercial interests. There are several challenges in terms of provision of authoritative unbiased information to publics to support their decision-making about personal genomic testing. In addition to crafting appropriate messages at a suitable comprehension level, there is also the challenge of considering a credible and intuitively accessible site and social media channel for the information that will reach the intended users.

In the discussion of the various purposes for which personal genomic testing is marketed, there was a range of views on the predictive capacity of genetic tests for traits such as sporting, musical abilities, as well as personality traits, with the majority of participants expressing doubts about the usefulness of genetic testing for these characteristics. Furthermore, some participants were concerned that technology may drive and potentially undermine personal and societal values, such as providing the opportunity to create designer babies. However, there was the perception of greater usefulness of health-related genetic information, even though for some obtaining this information could be anxiety-provoking. While about half of the participants said they would consider testing, most felt their motivation to be tested would be based on anticipation that they could obtain actionable health information relevant for themselves or their family, including information regarding genetic conditions that might be passed on to future children, in other words, carrier testing. Whether this would actually translate into behavior change, however, is a matter of ongoing debate [33]. Studies in the USA with consumers who have undergone personal genomic testing indicate minimal or moderate self-reported impact on lifestyle behavior change, such as diet and exercise [34–36], although there was greater sharing of information with doctors and some further test use [34, 36, 37].

Other motivations mentioned by the focus group participants for considering testing included an altruistic view of contributing to research, and forms of curiosity. In a systematic review of the literature on genomic testing in a range of contexts, altruism and curiosity were identified as elements of personal utility [38].

Overall, participants in our focus groups were cautious about the companies’ commercial stake in testing and their marketing practices. Those less keen on PGT cited concerns about negative impact of testing on privacy and insurance, leading to potential discrimination. In Australia, there have been calls recently for the government to provide closer oversight of the use of genetic and genomic information by the insurance industry, with concerns about their self-regulatory practices [39]. Insurers can request potential applicants of life insurance and other mutually rated products (but not health insurance) to disclose results from any genetic test, including results provided through personal genomic testing. A few examples of discrimination have been reported previously and the authors argue that government oversight is needed to ensure public confidence and trust, especially if benefits of genomic research and genomic medicine are to be realized [32]. It is unclear to what extent users of PGT have encountered discriminatory practices by insurers in Australia, where there is no moratorium on the use of genetic information by insurers, unlike in the UK and several European countries [39].

We acknowledge limitations of this exploratory qualitative study. Participants were self-selected and attendance at the focus groups was also constrained by their availability regarding time and locations at which the focus groups were held. The sample is not intended to be representative of the Australian public and, in general, participants were well-educated, with about one-third of the sample being university students, but a strength of the study is the inclusion of a wide range of ages. Experience with online PGT was limited and so views were expressed from the perspective of low awareness, and provide a snapshot in time. However, these attitudes serve as a useful counterpoint to those of early adopters of testing, and likely reflect the relatively low market availability and visibility of PGT in Australia at the time. Most recently, there has certainly been a rise in the advertising of DNA testing for ancestry purposes in the Australian media [20].

In conclusion, we present the findings from the first qualitative stage of a larger mixed-methods, multi-stage national study (Genioz) and provide exploratory data regarding views of Australians about PGT. Overall, awareness of these online tests was generally low at the time of conducting these focus groups, while their level of knowledge and understanding of genetics and genomics was varied and appeared to be drawn from several sources. Generally, participants’ views about genetics and genomics supported the contribution of environment and lifestyle to a person’s characteristics, including health. Attitudes to having PGT were mixed, with greater interest in health-related tests if there was an underlying motive; however, many participants expressed scepticism about the types of tests available and what might be done with that information. Findings will be used to inform a national survey and subsequent in-depth interviews with survey participants to gather views from a broader sample. In addition, findings will contribute to communication strategies to inform Australian policy makers and education resources for the public, with an emphasis on ensuring that language used is appropriate and relevant, and avoiding jargon such as “direct-to-consumer”.

## Electronic supplementary material

Table S1: Recruitment approaches

Table S2: Focus group schedule of questions

Figure S1: Word cloud of words used in response to the question “What springs to mind when I say the words ‘DNA’ or ‘gene’?”

Figure S2: Sources of information about genetics and DNA
