# Supplementary material for: Australians’ views on personal genomic testing: focus group findings from the Genioz study
Source: Eur J Hum Genet. 2018 Apr 30;26(8):1101–12. doi: 10.1038/s41431-018-0151-1 (PMC6057916; doi:10.1038/s41431-018-0151-1)
Supplement: Supplementary file 1 — Table S1: Recruitment approaches [file 41431_2018_151_MOESM1_ESM.docx]

**Supplementary Table S1. Recruitment approaches**

| **Form of advertisement** | **Distribution sources** |
| --- | --- |
| Hard copy posters | - Cafes in Sydney and Melbourne - Posted in local libraries - Community and leisure centres - Special interest societies (for example, genealogical society) |
| Email list | - Through personal networks of the research team, including university students - Special interest clubs (for example, a seniors travel club, University of the Third Age) |
| Online Facebook page posts | - Research institutes - Sport club Facebook page - Area/location-specific online notice boards in Victoria |
| Online advertisements | - Gumtree Australia (region-specific – for New South Wales and Victoria), a classified advertisement and community website |
