# Supplementary material for: Australians’ views on personal genomic testing: focus group findings from the Genioz study
Source: Eur J Hum Genet. 2018 Apr 30;26(8):1101–12. doi: 10.1038/s41431-018-0151-1 (PMC6057916; doi:10.1038/s41431-018-0151-1)
Supplement: Supplementary file 2 — Table S2: Focus group schedule of questions [file 41431_2018_151_MOESM2_ESM.docx]

**Supplementary Table S2. Focus group schedule of questions**

| Purpose of question | Questions and prompts |
| --- | --- |
| Round robin questions asked of each participant | In a few words, what springs to mind when you hear the words DNA or genes?  What about when I say the words ‘personal genomics’? |
| Transition questions | Let’s have a discussion about to what extent you think genes might play a role in…  *Prompts:*   - Health, physical characteristics, behaviour |
|  | How is it that you’ve come to these opinions?  Where do you get your information from?  *Prompts:*   - Media, TV, movies, newspapers, online, your doctor, family friends, formal education, Google, your gymnasium, an alternative health practitioner |
|  | How much confidence do you place in these as sources of accurate information? |
| Key questions | How much do you know about what’s involved in genetic testing? |
| - Scenarios/situations where personal genomics is used | Going back to personal genomics, what do you think the information from these tests might be used for?  *Prompts*:   - Fitness and athletic ability, nutrition, identity and genetic genealogy - Reproductive decisions, family relationships, predisposition to health and testing of children - Predisposition testing for health for themselves and testing of their grandchildren (as appropriate), genetic genealogy and pharmacogenomics |
|  | To what extent were you familiar with personal genomics before today?  *Prompts:*   - Have you heard of direct-to-consumer genetic testing? - Have you/any of your family/friends had a test like this? Or a genetic test for a medical condition? What was your/their experience? |
| - Benefits of personal genomics | What are some of the benefits of personal genomics you can think of?  *Prompts*:   - Think of personal benefits, benefits to family and benefits to society. |
| - Harms of personal genomics | What are some of the concerns around personal genomics you can think of?  *Prompts*:   - Think of personal, family and societal concerns. |
| - Interest in personal genomic testing | Now that we’ve had this discussion, do you think you might be interested in having any of these tests that we’ve talked about? Which ones? Pros and cons? |
