# Supplementary figures and images for: Australians’ views on personal genomic testing: focus group findings from the Genioz study
Source: Eur J Hum Genet. 2018 Apr 30;26(8):1101–12. doi: 10.1038/s41431-018-0151-1 (PMC6057916; doi:10.1038/s41431-018-0151-1)

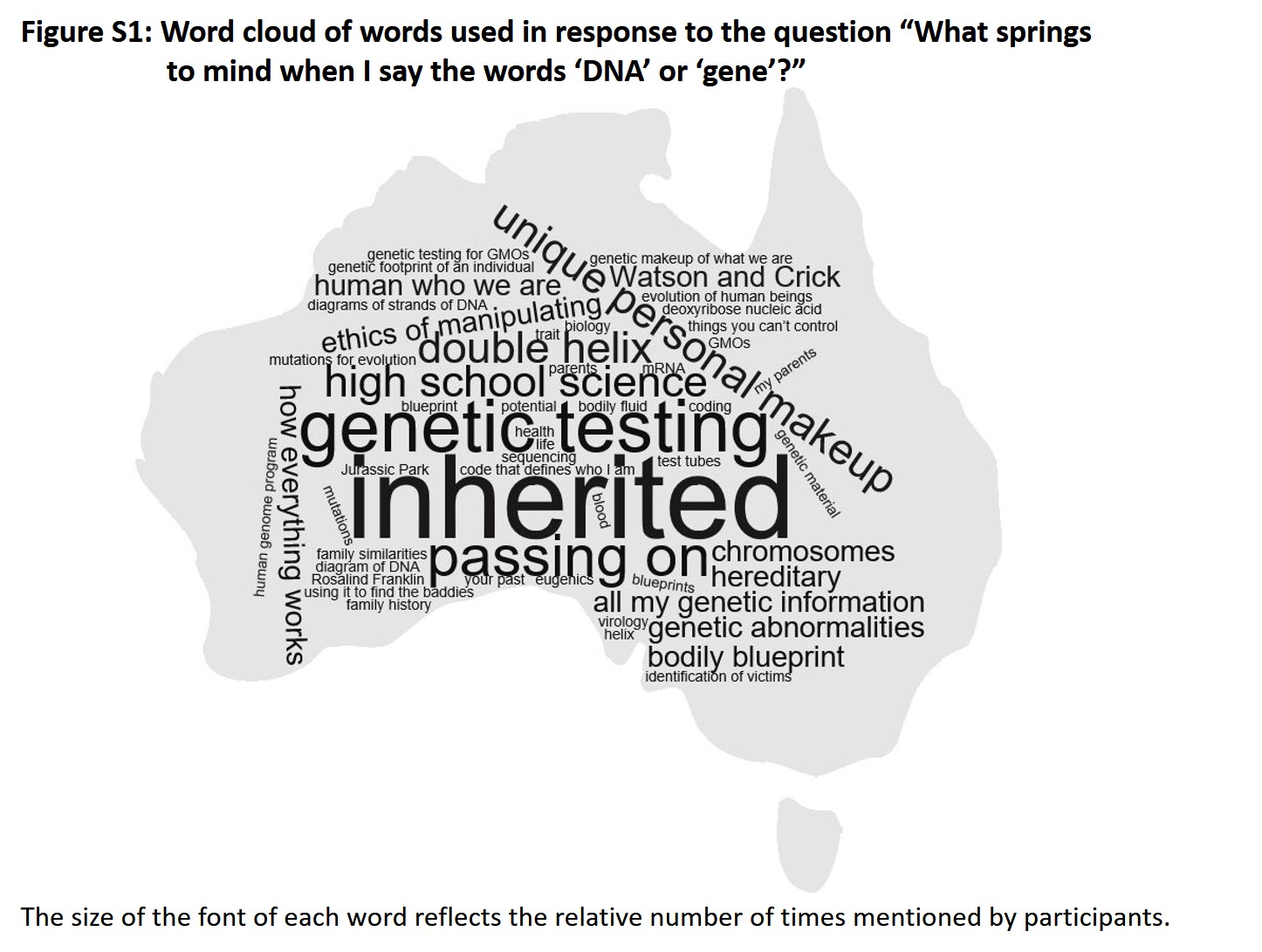

Supplement: Supplementary file 3 — Figure S1: Word cloud of words used in response to the question “What springs to mind when I say the words ‘DNA’ or ‘gene’?” [file 41431_2018_151_MOESM3_ESM.jpg]

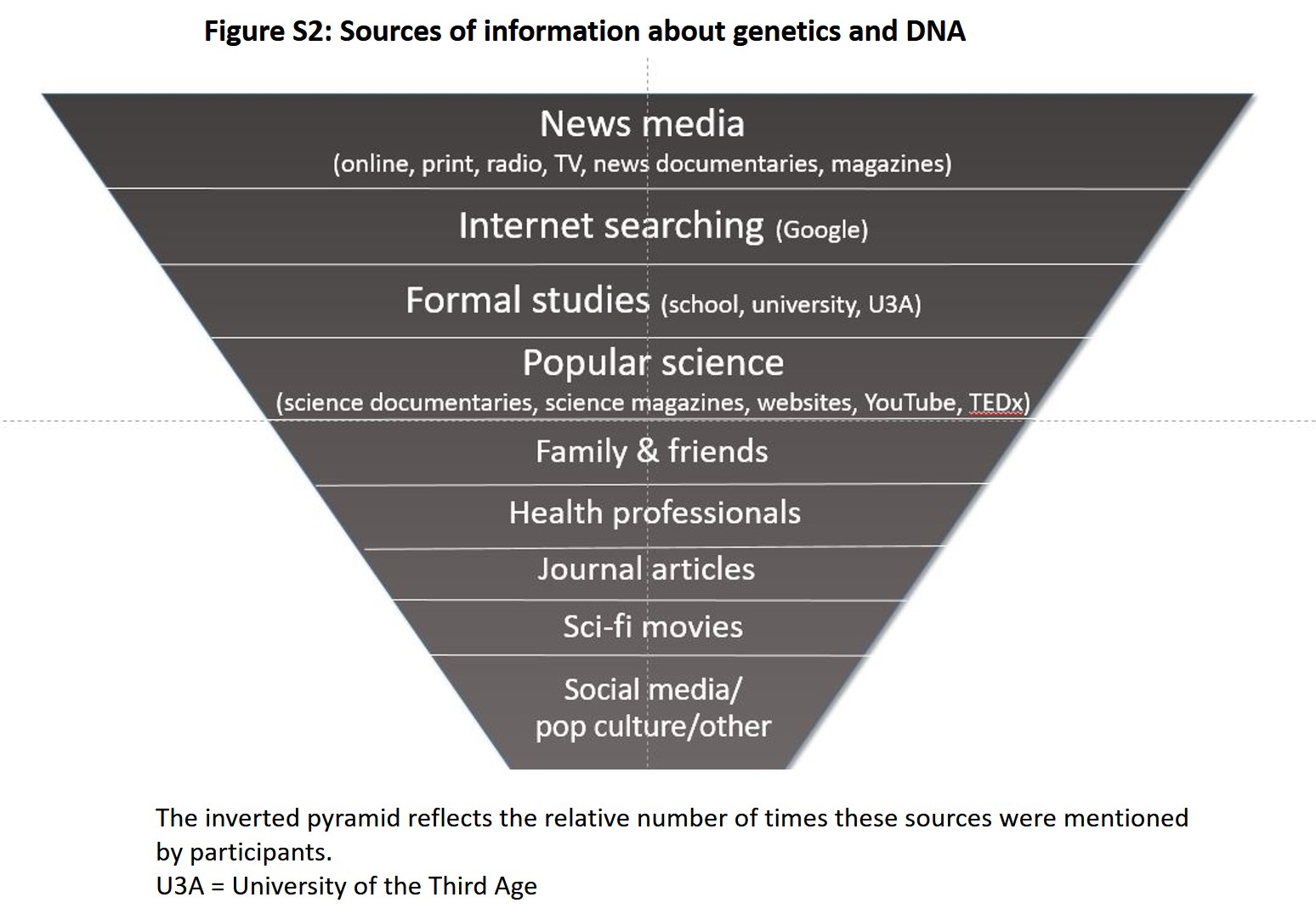

Supplement: Supplementary file 4 — Figure S2: Sources of information about genetics and DNA [file 41431_2018_151_MOESM4_ESM.jpg]
